# Draft Genome Sequence of *Methylophaga muralis* Bur 1, a Haloalkaliphilic (Non-Methane-Utilizing) Methylotroph Isolated from a Soda Lake

**DOI:** 10.1128/genomeA.01227-16

**Published:** 2016-11-03

**Authors:** Yuri A. Trotsenko, Maria N. Shmareva, Nina V. Doronina, Sergey V. Tarlachkov, Ildar I. Mustakhimov, Oleg V. Vasilenko

**Affiliations:** a G.K. Skryabin Institute of Biochemistry and Physiology of Microorganisms, Russian Academy of Sciences, Pushchino, Moscow Region, Russia; b Branch of Shemyakin and Ovchinnikov Institute of Bioorganic Chemistry, Russian Academy of Sciences, Pushchino, Moscow Region, Russia

## Abstract

The draft genome sequence of *Methylophaga muralis* strain Bur 1 (VKM B-3046T), a non-methane-utilizing methylotroph isolated from a soda lake, is reported here. Strain Bur 1 possesses genes for methanol and methylamine (methylamine dehydrogenase and N-methylglutamate pathway) oxidation. Genes for the biosynthesis of ectoine were also found.

## GENOME ANNOUNCEMENT

The genus *Methylophaga* currently includes 10 species with validly published names that are mostly moderately halophilic restricted-facultative methylotrophs that oxidize methanol with the subsequent assimilation of formaldehyde in the ribulose monophosphate (RuMP) pathway cycle. They are typically isolated from the marine environment and soda lakes (1) and make an important contribution to the biospheric cycle of carbon, nitrogen, and sulfur. Methylamine metabolism among *Methylophaga* spp. include both the methylamine dehydrogenase and N-methylglutamate (NMG) pathways. 

*M. muralis* Bur 1 is a moderately haloalkaliphilic methylotroph, isolated from the soda lake Khilganta, in Buryatia, Russia. DNA was disrupted by sonication, to obtain fragments with an average size of 400 to 500 bp (S2/E210 Focused-ultrasonicator, Covaris, USA), according to the manufacturer’s protocol. The draft genome of *M. muralis* Bur 1, determined by a whole-genome shotgun approach using Ion Torrent PGM (Thermo Fisher Scientific, Inc., USA) with a 400-bp sequencing kit and a 318 v2 chip. A total of 778,671 raw reads were assembled *de novo* into 130 contigs (75-fold peak coverage) using Newbler version 2.9 with the default parameters (454 Life Sciences Corporation, USA). The draft genome size is 2,846,920 bp with an average G+C content of 44.67%. The *N*_50_ contig is 76,869 bp, and the largest contig is 341,152 bp. Annotation was done using the Prokka version 1.11 program annotator (2) with the Barrnap version 0.5 plug-in (http://www.vicbioinformatics.com/software.barrnap.shtml).

The genome of *M. muralis* Bur 1 harbors 2,793 protein-coding genes, and one tmRNA, three rRNAs (one 5S, one 16S, and one 23S rRNA operon), and 39 tRNAs were identified. The gene cluster responsible for methanol oxidation (*mxaFJGIRSACKLDHED*) and genes involved in pyrroloquinoline quinone biosynthesis (*pqqBCDE*) were identified. Genes encoding enzymes of ectoine biosynthesis arranged in the typical haloalkaliphilic methylobacteria cluster *ectABC-ask* were identified (3). Genes encoding enzymes of the methylamine dehydrogenase (*mauABDEF*) and NMG pathway (*mgdABCD*, *mgsABC*, and *gmaS*) were detected. Nitrate reductase (*NapA*), two nitric oxide reductases (*NirB* and *NirD*), and genes of sulfur metabolism (*SoxYZ*) were detected.

Genes of tricarboxylic acid were identified, but α-ketoglutarate dehydrogenase, succinate dehydrogenase, and succinyl-CoA synthetase were absent. Genes encoding enzymes of the Entner-Doudoroff variant of the RuMP were detected. Also, among them, two copies of 3-hexulose-6-phosphate synthase (EC 4.1.2.43) were found. Key genes associated with the serine or the ribulose-bisphosphate pathway of C1 assimilation were absent, in agreement with enzyme assay data.

This whole-genome sequencing work is a new milestone for the taxonomy and evolution of the methylotrophs and their particular biochemical pathways. The additional bonus of the current research is using a type material.

### Accession number(s).

This whole-genome shotgun project has been deposited at DDBJ/EMBL/GenBank under the accession number MCRI00000000. The version described in this paper is the first version, MCRI00000000.1.

## References

[1] Boden R 2012 Emended description of the genus *Methylophaga* Janvier et al. 1985. Int J Syst Evol Microbiol 62:1644–1646. doi:10.1099/ijs.0.033639-0. 21890722

[2] Seemann T 2014 Prokka: rapid prokaryotic genome annotation. BioInformatics 30:2068–2069. doi:10.1093/bioinformatics/btu153. 24642063

[3] Reshetnikov AS, Khmelenina VN, Mustakhimov II, Kalyuzhnaya M, Lidstrom M, Trotsenko YA 2011 Diversity and phylogeny of the ectoine biosynthesis genes in aerobic, moderately halophilic methylotrophic bacteria. Extremophiles 15:653–663 doi:10.1007/s00792-011-0396-x. 21971967

